# Revolutionizing Care: Unleashing the Potential of Digital Health Technology in Physiotherapy Management for People With Cystic Fibrosis

**DOI:** 10.2196/55718

**Published:** 2024-07-15

**Authors:** Lisa Morrison, Zoe Louise Saynor, Alison Kirk, Lisa McCann

**Affiliations:** 1 West of Scotland Adult Cystic Fibrosis Unit, Queen Elizabeth University Hospital, Glasgow, United Kingdom; 2 Department of Computer and Information Sciences, University of Strathclyde, Glasgow, United Kingdom; 3 School of Sport Health and Exercise Science, University of Portsmouth, Portsmouth, United Kingdom; 4 Cystic Fibrosis Unit, University Hospital Southampton NHS Foundation Trust, Southampton, UK; 5 Department of Psychological Sciences and Health, University of Strathclyde, Glasgow, United Kingdom

**Keywords:** cystic fibrosis, physiotherapy, digital technology, telehealth, cystic fibrosis transmembrane regulator modulators, telemedicine, digital health technology, DHTs, digital health, physical therapy, physical activity, exercise, monitoring, physiotherapists, user, experience, remote, virtual care, consultation, consultations, eConsultations, preferences, digital divide, access, accessible, accessibility, attitude, perception, attitudes, opinion, perceptions, perspectives, eHealth, online health, therapy

## Abstract

This viewpoint paper explores the dynamic intersection of physiotherapy and digital health technologies (DHTs) in enhancing the care of people with cystic fibrosis (CF), in the context of advancements such as highly effective modulator therapies that are enhancing life expectancy and altering physiotherapy needs. The role of DHTs, including telehealth, surveillance, home monitoring, and activity promotion, has expanded, becoming crucial in overcoming geographical barriers and accelerated by the recent pandemic. Physiotherapy, integral to CF care since 1946, has shifted toward patient-centered approaches, emphasizing exercise training and a physically active lifestyle. The reduction in inpatient admissions due to highly effective modulator therapies has led to increased home care and online or electronic consultations, and DHTs have revolutionized service delivery, offering flexibility, self-management, and personalized care options; however, there is a need to comprehensively understand user experiences from both people with CF and physiotherapists. This paper highlights the essential exploration of user experiences to facilitate clinician adaptation to the digital requirements of modern clinical management, ensuring equitable care in the “future hospitals” arena. Identifying research gaps, this paper emphasizes the need for a thorough evaluation of DHT use in CF physiotherapy education, training, and self-monitoring, as well as the experiences of people with CF with online or electronic consultations, self-monitoring, and remote interventions. Online group exercise platforms address historical challenges relating to infection control but necessitate comprehensive evaluations of user experiences and preferences. Future-proofing DHTs within the physiotherapy management of CF demands a shift toward full integration, considering stakeholder opinions and addressing barriers. While DHTs have the potential to extend physiotherapy beyond the hospital, this paper stresses the importance of understanding user experiences, addressing digital poverty, and working toward more equitable health care access. A flexible approach in the “future hospital” is advocated, emphasizing the need for a nuanced understanding of user preferences and experiences to optimize the integration of DHTs in CF care.

## Introduction

Cystic fibrosis (CF) is a chronic, autosomal recessive, life-limiting, multisystem disease, historically leading to respiratory failure and premature death [1]. Chest physiotherapy (airway clearance techniques) to enhance secretion clearance has been a cornerstone of CF physiotherapy, with self-management and guided management being the focus of care as people with CF develop and their disease dictates different approaches. Advancements in the clinical management of CF, including the introduction of highly effective modulator therapies (HEMTs), have positively impacted life expectancy [1,2]. Consequently, physiotherapy management of CF and the specialist CF physiotherapist must adapt [3].

There is a growing body of evidence supporting examples where physiotherapy has benefited from digital health technologies (DHTs), primarily existing in the management of musculoskeletal [4] or neurological issues [5,6]. Here, specific exercise, virtual reality, and gaming have positively influenced rehabilitation; however, this new innovative technology currently does not exist in CF physiotherapy management. DHTs have been used in CF for some time, particularly in areas of geographical diversity, with online or electronic consultation and monitoring therapies becoming increasingly commonplace [7]. DHTs have been used in chipped nebulizers monitoring adherence to therapeutic regimes [8] and home spirometers, alongside other wearables and mobile apps (eg, the Project Breathe patient-driven symptom reporting [9]). There has, however, been limited research into the evaluation of online or electronic physiotherapy interventions in CF, the implementation of DHTs, and their effectiveness within the physiotherapy management for CF.

Online or electronic physiotherapy in CF could facilitate more than symptom monitoring, extending to simple exercise testing, remote physical activity, and exercise opportunities, as well as implementing measures to influence adherence and the prompt management of symptoms. While online or electronic consultations may be more convenient for some people with CF and reduce the risk of cross infection, not all people with CF will benefit from reducing the frequency of in-person consultations.

## The Changing Role of Physiotherapy Within CF Care

Physiotherapists, originally involved in CF care for chest clearance in 1946, now participate in a global clinical and research network, developing national and international clinical guidance and standards of care [3,10-12]. While global standards of clinical care exist, there will be variations in the implementation of these and DHTs due to socioeconomic factors, availability of infrastructure, and accessibility in health care settings and beyond [13,14]. Irrespective of these challenges, an awareness of data storage, accessibility, and safety of data is essential, and the physiotherapist must be mindful of these factors.

CF physiotherapy has progressed to a more active, patient-centered approach to clinical care [11,12]. This still includes airway clearance techniques and assessment of respiratory and nonrespiratory manifestations (eg, musculoskeletal and sinuses) and, with a rising prevalence of increase in weight leading to obesity [15] and cardiovascular diseases [16], an ever-increasing involvement in the promotion of exercise testing, training, and physical active promotion. The reduction in inpatient admissions following HEMT has enabled an increase in home care and online or electronic consultations, reducing reliance on hospital services, mitigating cross infection risks, and reducing travel to hospital.

Online or electronic consultations assess people with CF remotely, with mobile devices monitoring symptoms, assessing pulmonary function, and patient-reported outcomes, as well as promotion of physical activity [17,18]. These have been well received, but with variable compliance due to competing demands impacting overall uptake [17]. Several online or electronic platforms, some led by physiotherapists, offered education and training to people with CF and health care professionals, enabling widespread delivery of information and resources, with the potential for standardized data collection and optimized quality care [17,19-22].

Self-monitoring, particularly spirometry, has been explored, with physiological data and symptom recognition proposing earlier identification of pulmonary exacerbation [9,23-25]. Self-monitoring, however, may be less accurate, leading to undetected worsening of health status [23,24]. Despite suggestions that self-monitoring is well used [24,26], uploading of digital data is poorly adhered to, and collecting data should be optimized based upon clinical usefulness [23]. Cox et al [27] highlighted in a systematic review that >50% of participants were noncompliant with data entry, with data upload considered burdensome, potentially intrusive, and a barrier to maintainability. Exploration of opportunities for continuous monitoring or passive uploading of data (as occurs with some wearables [9]) may reduce the burden on people with CF and positively influence their use of devices. Improving the accuracy of self-monitoring and symptom monitoring using DHTs may facilitate swifter directed access to relevant professionals providing individually tailored treatments, facilitating personalized discussion and, ultimately, leading to more user-driven outcomes (NCT04798014) [28,29].

Following the introduction of HEMT, many clinical outcomes observed in people with CF with access have improved, such as fewer pulmonary exacerbations, improved lung function, and exercise tolerance [30]. The physiotherapists’ role in exercise testing, training, and promoting a physically active lifestyle is well researched [31-34] and remains central to the maintenance and optimization of health in people with CF [12]; however, there are no specific CF-related physical activity guidelines [35]. Uptake and adherence to physical activity programs in people with CF is poor [19], and this occurs irrespective of remote delivery [36]. Physical activity is central to the CF physiotherapist’s role; however, segregation requirements historically rendered group activities unachievable. Physical activity platforms have enabled physiotherapists to deliver online group exercise [23], both live and on demand [20], and have been shown in other chronic illnesses to provide solutions to remotely support physical activity and emotional well-being, and improve quality of life [37]. Online group activities allow people with CF to experience peer support [17,20,27], physiotherapy supervision, and education pertinent to their health [20,38]. Despite the anticipated positives of this, significant dropout and discontinuation in some centers have occurred. It is important to evaluate reasons for this and engage with people with CF to identify user opinions for future online physical activity provision.

## The Use of DHTs in the Physiotherapy Management of CF

The benefits of DHTs in CF care include reducing cross infection [39] and enabling interprofessional team management in areas with diverse geographic distances [7]. People with CF have responded positively to remote consultation, online exercise provision, and monitoring [8,12,13].

Airway clearance quality has been shown to have a greater impact on respiratory function than quantity and frequency [40,41]. The integration of DHT using pressure sensors embedded in devices may influence physiotherapy assessment and treatment delivery. Using DHTs to guide, counsel, and facilitate goal attainment may enable individualized physiotherapy care for people with CF, offering a flexible approach to modern management, enhancing adherence, and impacting clinical outcomes important to people with CF. For example, the use of wearables and supportive messaging from physiotherapists demonstrated a prolonged and positive change in step count and exercise capacity in adults with CF [42]. Assessment of data derived from online or electronic consultation and self-monitoring can guide assessment of what has worked well and what should perhaps be discontinued. This will include the evaluation of digital literacy skills and acceptance by people with CF in using DHT to access their health care teams effectively and appropriately.

The role of telehealth for exercise testing has been shown in other diseases to offer a viable alternative to some in-person testing [43] and requires further exploration in CF. To date exercise testing in people with CF has not been researched in a online or electronic capacity but could support centers with limited or no access to in-house exercise testing facilities.

## Can We Future-Proof and Optimize the Use of DHTs Within the Physiotherapy Management of CF?

DHTs are not yet fully integrated into CF management locally or globally [44] and are often considered an “add-on.” Electronic patient records are widely used but have not fully replaced conventional written records for all consultations. Opinions of health care teams and people with CF are essential to strengthen the implementation and maintainability of any future DHTs in routine care [24]. Further research into barriers and facilitators for maintained use of DHTs will support long-term digital plans [45,46]. The optimization of current data uploading applications and platforms to ensure that they are clinically useful for both the user and the stakeholders must occur, including support for training and education when using DHT [44].

There are numerous frameworks (eg, RE-AIM [Reach, Effectiveness, Adoption, Implementation, and Maintenance] [47] and NASSS [nonadoption, abandonment, scale-up, spread, sustainability] [48]) developed specifically for identifying interacting influences dictating the success or failure of a system [46,49-51]. The recent analysis of the physical activity in people with CF [19] recognized that frameworks offer reasons for nonengagement, with respect to relevance and user satisfaction with interventions and associated technology. Future research should apply these frameworks, exploring how to improve the uptake and use of DHT [52].

Implications of DHT should be considered, as changing one aspect may influence (positively or negatively) other areas of care [53], and the introduction of DHTs in managing children and adolescents with CF will be significantly different to adults and those with multimorbidities. Some people with CF are digital natives, growing up with an appreciation of DHTs; others have lower levels of digital literacy and trust in digital services and, consequently, the uptake of opportunities to influence their health using this technology may be lower [54,55]. DHTs could negatively impact the “personal” feel of a consultation, leaving people with CF feeling that they are no longer “known” to their clinical care team with respect to their wider societal issues [56].

## Conclusions

DHTs present exciting potential for physiotherapy management in CF. Online or electronic consultations, online physiotherapy (including physical activity and exercise training), and remote monitoring may, however, not be desirable, available, or appropriate for everybody. We urgently need to understand the experience of early implementers, the enablers of success, and the needs of the CF community to better inform equitable use. We must ensure this does not create a digital divide, as digital poverty continues to exist, impacting digital and health literacy, use, and practical application of DHT. We must ensure online or electronic consultations meet the requirements of those accessing them. To ensure “no one is left behind” and optimize care for people with CF, we need to challenge the unsupportable “one-size-fits-all” approach. This involves a flexible infrastructure supporting the future physiotherapy management of people with CF, based on patient experience–related reported outcomes allowing refinement and delivery of an optimal and individualized service.

## Abbreviations

CF: cystic fibrosis
DHT: digital health technology
HEMT: highly effective modulator therapy
NASSS: nonadoption, abandonment, scale-up, spread, sustainability
RE-AIM: Reach, Effectiveness, Adoption, Implementation, and Maintenance
